# Feeding Immunity: Physiological and Behavioral Responses to Infection and Resource Limitation

**DOI:** 10.3389/fimmu.2017.01914

**Published:** 2018-01-08

**Authors:** Sarah A. Budischak, Christina B. Hansen, Quentin Caudron, Romain Garnier, Tyler R. Kartzinel, István Pelczer, Clayton E. Cressler, Anieke van Leeuwen, Andrea L. Graham

**Affiliations:** ^1^Department of Ecology and Evolutionary Biology, Princeton University, Princeton, NJ, United States; ^2^Department of Veterinary Medicine, University of Cambridge, Cambridge, United Kingdom; ^3^Department of Ecology and Evolutionary Biology, Brown University, Providence, RI, United States; ^4^Department of Chemistry, Princeton University, Princeton, NJ, United States; ^5^School of Biological Sciences, University of Nebraska, Lincoln, NE, United States; ^6^NIOZ Royal Netherlands Institute for Sea Research, Department of Coastal Systems, and Utrecht University, Texel, Netherlands

**Keywords:** *Trichuris muris*, resource–immune trade-offs, compensatory feeding, DNA metabarcoding, nuclear magnetic resonance spectroscopy metabolite profiling, rewilding mice

## Abstract

Resources are a core currency of species interactions and ecology in general (e.g., think of food webs or competition). Within parasite-infected hosts, resources are divided among the competing demands of host immunity and growth as well as parasite reproduction and growth. Effects of resources on immune responses are increasingly understood at the cellular level (e.g., metabolic predictors of effector function), but there has been limited consideration of how these effects scale up to affect individual energetic regimes (e.g., allocation trade-offs), susceptibility to infection, and feeding behavior (e.g., responses to local resource quality and quantity). We experimentally rewilded laboratory mice (strain C57BL/6) in semi-natural enclosures to investigate the effects of dietary protein and gastrointestinal nematode (*Trichuris muris*) infection on individual-level immunity, activity, and behavior. The scale and realism of this field experiment, as well as the multiple physiological assays developed for laboratory mice, enabled us to detect costs, trade-offs, and potential compensatory mechanisms that mice employ to battle infection under different resource conditions. We found that mice on a low-protein diet spent more time feeding, which led to higher body fat stores (i.e., concentration of a satiety hormone, leptin) and altered metabolite profiles, but which did not fully compensate for the effects of poor nutrition on albumin or immune defenses. Specifically, immune defenses measured as interleukin 13 (IL13) (a primary cytokine coordinating defense against *T. muris*) and as *T. muris*-specific IgG1 titers were lower in mice on the low-protein diet. However, these reduced defenses did not result in higher worm counts in mice with poorer diets. The lab mice, living outside for the first time in thousands of generations, also consumed at least 26 wild plant species occurring in the enclosures, and DNA metabarcoding revealed that the consumption of different wild foods may be associated with differences in leptin concentrations. When individual foraging behavior was accounted for, worm infection significantly reduced rates of host weight gain. Housing laboratory mice in outdoor enclosures provided new insights into the resource costs of immune defense to helminth infection and how hosts modify their behavior to compensate for those costs.

## Introduction

**Figure 1:**
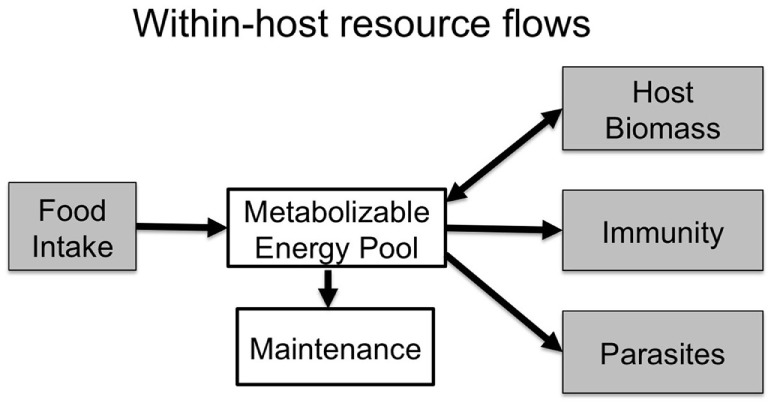
Within an infected host, resources are metabolized and allocated to baseline maintenance costs. Remaining resources are put toward immunity and host biomass, or are captured by parasites to use for their own growth and reproduction. Resources, therefore, must trade-off between these competing demands unless hosts are able to increase the quality or quantity of food intake to compensate for those costs.

Ecologists have long-studied energy and nutrient flows in ecosystems to understand their function and how they may respond to environmental changes (1–4). In ecological communities, these flows are often a core currency of species interactions in food webs. Parasite–host interactions represent a trophic interaction in their own right, and elucidating resource flows within infected hosts can reveal crucial processes that determine immunity and infection outcomes. Resources ingested by hosts must first be metabolized and used for maintenance (i.e., baseline metabolism), and can subsequently be used for host growth (including growth of immune cells), or be diverted to parasite growth and reproduction (Figure 1).

Resource flow models also capture the total cost of infection, including nutrients going to immunity, parasites, and host tissue repair. By modeling the priority of resource allocation to the host’s immune system vs. resources captured by parasites, Cressler et al. (5) illustrated how increasing resource acquisition can have qualitatively different effects on host immunity and parasite loads: the immune priority scenario (i.e., when additional resources go first into immune system components) quickly clears infections, while the parasite priority scenario (i.e., additional resources are first nabbed by the parasite), results in chronic infections. At molecular and cellular levels, immunologists are increasingly describing how nutrients and metabolites affect particular immune pathways (6–8). For example, receptors for glucose and leptin, a signal of body fat (9), are found on T- and B-lymphocytes, macrophages, neutrophils, and natural killer cells and can stimulate inflammatory responses [reviewed in Ref. (6, 10)]. While great progress has been achieved in understanding how nutrition affects immunity (7), scaling-up our understanding of resource–immune interactions from molecules and cells to entire organisms and populations remains challenging (11). Here, we use semi-natural enclosures to investigate resource flow through parasitized hosts by examining trade-offs among immunity, host condition (protein and fat stores), and parasite growth and survival under two levels of resource availability.

Because of their tractability and the plethora of tools available for studying immune pathways, laboratory (lab) mice have been integral in building our basic understanding of immunology, as well as how parasite-infected hosts respond to energy, macronutrient, and micronutrient limitation (12–17). However, when scaling to organism-level questions of nutrition and resource flows during infection, it is becoming apparent that lab mouse experiments do not recapitulate some critical biological features (Table 1). The *ad lib*, energy-rich, readily accessible resource conditions of lab mice differ from those of most human and wildlife populations. Additionally, ties between immunity, metabolism, and the gut microbiome are increasingly recognized (18, 19), and the low diversity microbiomes and low activation state of T cells of lab mice most closely resemble neonates (20) and diverge widely from wild mice (21). Furthermore, gastrointestinal (GI) helminths also play a role in training and modulating immunity (22, 23), with increasing risks of allergic and autoimmune conditions in human populations devoid of their coevolved worms (24). Thus, this and other studies of helminth infection in rewilded mice^1^ provide the opportunity to study how these key components of immunological regulation interact to affect the health of humans and other animals.

**Table 1:** Studying laboratory mice in semi-natural enclosures provides a tractable experimental system that recapitulates more aspects of wild systems than traditional laboratory experiments while avoiding confounding complexities such as unknown exposure histories and coinfections.

	Lab	Enclosures	Wild
Host genotype, age, sex	+ selectable, − not diverse	+ selectable, − not diverse	Natural but added source of variation
Previous exposure	Controlled	Controlled	Unknown and can affect immune investment
Coinfections	Controlled	Controlled	Unknown and can affect immune investment
Thermoregulation	Artificial constant temperature	Natural	Natural
Foraging	Only chow, accessed with minimal foraging effort	Chow accessible with moderate effort, natural forage	Natural forage requiring greater energetic investment to acquire
Diet manipulation and feeding behavior	Manipulatable but cannot track individual feeding	Manipulatable and can track individual feeding	Limited to providing supplementary food, cannot track individual feeding
Predators and competitors	None	Excluded	Natural
Reproduction	None^a^	None^b^	Natural
Seasonality	None	Present	Present
Microbiome	Limited (20)	More diverse (See text footnote 1)	Natural
Immunological tools	Widely commercially available	Widely commercially available	Limited, more tools available for species closely related to lab and veterinary animals

*^a^Unless breeding pairs are purposely put together*.

*^b^None if housed in single-sex enclosures, but possibly reproduce if both sexes are cohoused*.

Fortunately, recent studies offer a promising compromise between realism and tractability for studying immune–resource interactions in lab mice. For example, immune traits of lab mice can be made more representative by generating more diverse microbiomes or exposure histories in the laboratory (20, 25). An even greater degree of realism can be achieved by putting lab mice in outdoor enclosures (Table 1) that arguably mimic aspects of their evolutionary history as commensals of humans engaged in agriculture (26). Outdoors, lab mice develop more diverse microbiomes, elevated T cell responses and higher parasite loads compared with indoor lab mouse controls (See text footnote 1). Additionally, in outdoor enclosures, mice experience more natural variation in activity and thermo-regimes that may make trade-offs between immunity and other physiological processes more apparent than under lab conditions (Table 1).

Host behavior is central to scaling-up immune–resource interactions from the cellular to organismal level. Mice on low-quality (i.e., protein) diets may consume a greater quantity of chow, which can alter their body fat composition and immune profiles (27). From livestock studies, we know that GI nematodes, or host immune responses to them, can reduce host appetite, decreasing the energy budget the host has to allocate to defense, repair, and other physiological functions (28). Reciprocally, hosts can alter foraging during infection by consuming medicinal plants (29) or by increasing foraging to mitigate costs of infection and immunity (30). In free-ranging populations, increased foraging may come with increased energetic costs, parasite exposure, social stress, and predation risks (11, 28, 31, 32). Thus, assessing feeding behavior is critical for understanding how organisms respond to resource limitation and the resultant fitness consequences (7).

Using a semi-natural field system (See text footnote 1), we manipulate resource availability to examine the resource costs of infection and immunity. Our goal was to investigate the costs of infection (including resources diverted to parasites and immunity) and to learn how hosts may use foraging behavior to mitigate those costs in lab mice (C57BL/6) infected with the GI nematode *Trichuris muris*. *T. muris* is a whipworm that lives in the cecum, and is a congener of the parasite *Trichuris trichiura* that infects over 450 million people worldwide (33). To assess how hosts respond to infection under resource limitation, we manipulate levels of dietary protein, which is known to have strong effects on host immune defenses (7, 17, 34–37). We might expect mice fed the high-protein diet to have stronger immune responses and lower parasite loads than those on the low-protein diet. Alternatively, mice on the high-protein diet could tolerate infection while mice on the low-protein diet resist (17), which would turn the expected observation around and show stronger immune responses and lower parasite loads in the low-protein treatment. At the individual level-scale, we track whether mice compensate for potential joint costs of infection or a low-protein diet by altering foraging behavior. We predict that there will be trade-offs between food intake, investment in immunity, and parasite load (Figure 1). Infected mice may either feed less due to infection-induced inappetence (28, 38, 39), or feed more to compensate for costs of infection and immunity. Our findings do indeed suggest complex repercussions of behavioral changes for the flow of resources into infection and immunity.

## Materials and Methods

### Experimental Design

**Figure 2:**
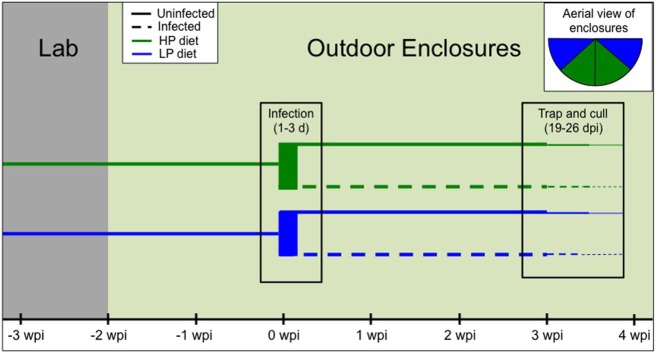
Timeline of the experimental design. First, mice were randomly assigned to diet treatment and cohort [−3 weeks postinfection (wpi)]. Diets were provided to the second cohort 2 days later, but since both groups subsequently followed the same timeline, only one cohort is depicted for clarity. After 10 days in the lab (−2 wpi), all mice were moved to four outdoor enclosures (*n* = 22/enclosure). After 2 weeks, 16 mice per enclosure were trapped and infected with 200 *T. muris* eggs over the course of 1–3 days. Final trapping and culling occurred around 3–4 wpi (19–26 days postinfection). Inset shows an aerial view of the enclosures by diet treatment, and infected and uninfected mice were cohoused.

Our experiment to examine trade-offs between infection, immunity, and within-host resources (i.e., diet quality) included four treatment groups: high-protein infected, high-protein uninfected, low-protein infected, and low-protein uninfected. Eighty-eight female C57Bl/6 mice aged 5–6 weeks were obtained from Jackson Laboratories and individually identified with both ear tags and RFID tags (see below). All animal care was conducted in accordance with protocols approved by the Princeton University Animal Care and Use Committee (Protocol no. 1982-14). Mice were housed in groups of five in the laboratory and randomly assigned to the two diet treatments as well as two cohorts that were staggered by 2 days for logistical purposes (Figure 2). The high-protein (HP; 20%; Envigo Teklad Custom Diet TD.91352) and low-protein (LP; 6%; Envigo Teklad Custom Diet TD.90016) diets had the same energy density (3.8 kcal/g) and micronutrient composition. The typical chow fed to lab mice (e.g., PicoLab^®^ Rodent Diet 20) has very similar composition to the HP diet. For 10 days mice were fed the assigned diets in the lab while temperature and light cycles were gradually altered to mimic outdoor conditions (June/July in New Jersey, USA: 26 ± 1°C with a 15-h light–9-h dark cycle; Figure 2).

Next, the 22 mice in each diet–cohort combination were transported to four outdoor enclosures (Figure 2), two of which contained the HP chow and the other two the LP chow (Figure 2 inset). The enclosures are replicate pens of approximately 180 m^2^, fenced in by zinced iron walls extending 1.5-m high and 80-cm deep, and topped with electric fencing and reflective aluminum pans to deter ground and aerial predators, respectively (See text footnote 1). Diets were provided *ad libitum* at feeding stations monitored by two RFID readers to track each individual’s time spent feeding. The natural environment could serve as an additional source of food (e.g., berries, seeds, insects). Each enclosure provided two watering stations inside a small (180 cm × 140 cm × 70 cm) straw-filled shed (See text footnote 1). Mice were weighed at the start of the experiment, the day of release, and approximately weekly thereafter as they were trapped overnight in the outdoor enclosures using chow-baited Longworth traps. At each weekly trapping, fecal samples were collected and blood samples were taken *via* shallow cutaneous tail snips into heparinized capillary tubes.

Mice were acclimated to the enclosures for 2 weeks (14–17 days) prior to *T. muris* infection (Figure 2). A dose of 200 embryonated *T. muris* eggs (strain E) was then given *via* oral gavage to the first 16 mice trapped per enclosure. If more than 16 mice were trapped on a given day, individuals were randomly assigned to infection treatment. In total, 29 mice on the HP diet and 31 mice on the LP diet were infected. Thus, infected and uninfected animals were cohoused in the same enclosures. The remaining mice (15 HP, 13 LP) served as uninfected controls. Nematode infection could not be transmitted between mice assigned to different treatments in the shared enclosures due to the long life cycle of *T. muris* [>28 days to maturity (40)], and the relatively short duration of the experiment [<26 days postinfection (dpi)].

Approximately 3 weeks postinfection (mean ± SE: 21.5 ± 1.7 dpi), range: 19–26 (dpi), all mice were trapped, weighed, and transported back to the laboratory (Figure 2). Mice were anesthetized via isoflurane inhalation followed by terminal cardiac puncture. Cardiac blood was drawn from each mouse, spun in a heparinized tube, and plasma was separated and stored at −80°C. During necropsy, mesenteric lymph nodes (MLNs) were excised for cell culture (see below). Finally, to determine carcass weight, all major organs (except the brain) were removed and weighed. The spleen, large intestine (emptied of contents), and cecum were separated and weighed. Ceca were frozen for later dissection and parasite enumeration.

### Quantifying Feeding Behavior

Individual-level feeding behavior was assessed using a custom-built monitoring system (Datasheet S1 in Supplementary Material). To track feeding behavior, Radio Frequency IDentification tags (RFID; 8 mm × 1.4 mm FDX-B “Skinny” PIT Tag, Oregon RFID, Portland, OR, USA) were injected subcutaneously. When in the vicinity of an antenna, the RFID tag emits a unique string of numbers that serves as an individual’s identifier (i.e., RFID number). In each enclosure, Feeding Event Tracking Apparatuses (FETA) with RFID antennas were deployed. The FETA transmit a signal to an Event Acquisition and Reporting System (EARS), which automatically compiles the data produced by the FETA (FETA number, RFID number, and timestamp). Two FETA were placed in sequence and connected on one end to the sole entrance to the chow hopper.

The directionality of mouse movement through the sequential FETA boxes was used to determine when mice were in the feeder. Specifically, mice were inferred to be in the feeder for the duration of time between two EARS readings on the FETA nearest the chow hopper. Intervals <5 s in duration (7.5% of visits) were removed because they allowed insufficient time to feed. Intervals greater than 2 h were also removed because mice were likely not eating for such a long duration and this small fraction (<0.1%) of visits skewed feeding time distributions. Intervals when chow was not available in the feeder (i.e., during trapping sessions) were also dropped. Next, total time feeding was summed per mouse, then divided by the number of days each mouse was in the enclosure because some mice were in the enclosures for longer than others (35–40 days depending on when they were trapped). This produced a comparable measurement of time spent feeding (min) per experiment day. Mice that lost their RFID tags (*n* = 6) and two individuals that did not eat at the feeder (working RFIDs but stopped visiting feeder) were excluded from feeding behavior analyses. Chow consumption was monitored by weighing each chow hopper every time it was removed for trapping as well as before and after refilling (i.e., every 1–3 days).

### Immune Response Measurements

Following established protocols (See text footnote 1, 41), MLN tissue was excised during necropsy, prepared into single-cell suspensions at a density of 5 × 10^6^ cells/mL, stimulated with *T. muris* antigen at 5 µg/mL, and cultured for 48 h. Supernatants were collected and analyzed in duplicate for interleukin 13 (IL13), interleukin 10 (IL10), interleukin 17 (IL17), and interferon gamma (IFNg) using half-reactions of Beckton Dickinson Cyometric Bead Array Mouse/Rat Soluble Protein Flex Set system (BD Biosciences, Oxford, UK) and an LSRII flow cytometer (BD Biosciences) (See text footnote 1). Concentrations were analyzed with the FCAP Array software (version 3.0.1, BD Biosciences).

Worm-specific IgG1 titers were measured using sandwich enzyme-linked immunoassays (ELISA). Ninety-six-well plates were coated with *T. muris* excretory-secretory (ES) antigen at a concentration of 5 µg/mL in carbonate buffer (0.06 M, pH = 9.6) with 2% bovine serum albumin (BSA). Plates were then blocked overnight with 2% powdered milk in carbonate buffer. After a 2-h incubation at 37°C, plates were washed three times with *tris*-buffered saline-Tween (TBST). Plasma samples were added and serially diluted from 1:50 to 1:6,400 with TBST and incubated for 2 h at 37°C. After washing five times with TBST, horseradish peroxidase (HRP) conjugated IgG1 antibody (1:4,000 in TBST) was added. Following a 1-h incubation at 37°C, plates were washed five times and Substrate ABTS (Sigma-Aldrich) was added. Plates were developed for 20 min at 37°C, then stopped with a 1% sodium dodecyl sulfate solution. Absorbance was measured at 405 nm using a Multiscan™ GO spectrophotometer (Thermo Scientific). Titers were determined by the dilution at which absorbance exceeded 4 SDs above background, defined as the plate-wide average absorbance of the two most dilute concentrations of each sample.

To quantify total IgG, a mouse antibody IgG pair was purchased from StemCell Tech (Catalog no. 01998C). Plates were coated at 1 µg/mL 50 μL per well with carbonate buffer (pH 9.6) overnight at 4°C. Plates were blocked for an hour with TBST 20 with 0.1% BSA at 37°C, then washed. Preliminary assays revealed that IgG levels were quite high, so to fall in the range of the standards, samples were diluted 1:81,920 in TBST containing 0.1% BSA. After incubation for 2 hat 37°C and washing, a secondary antibody was applied at a concentration of 1:1,000 in TBST with 0.1% BSA. Plates were incubated at 37°C for 1 h then washed. Next, we added 100 µL/well of p-nitrophenyl phosphate (pNPP) substrate to all wells. Finally, plates were incubated for 30 min and read at 405 nm. Concentrations were calculated in comparison to a plate-specific standard curve (all *R*^2^ > 0.999).

### Parasite Quantification

Caeca were cut open longitudinally and examined under a dissecting microscope by an observer blind to infection status. Worms were isolated by scraping the gut mucosa into a series of clean petri dishes of water. After enumeration, worms were stored in 70% ethanol for subsequent length measurements. Each worm was photographed under a dissecting scope (2–4× power) and ImageJ (version 1.49, NIH, USA) was used to measure total length.

### Condition and Nutritional Measurements

Plasma albumin concentrations were measured colorimetrically. QuantiChrom BCG albumin assay kits (BioAssays) were used following the manufacturer’s instructions. Briefly, 5-µL duplicates of each standard and sample (diluted 1:2 with ultrapure water) were mixed with 200 µL of BCG reagent. Plates were incubated at 23°C for 5 min before being read at 620 nm. Concentrations were determined in comparison to a standard curve run in duplicate (*R*^2^ = 0.997).

Plasma leptin concentrations were analyzed using a RayBio^®^ Mouse Leptin ELISA kit following manufacturer’s instructions (RayBiotech, Norcross, GA, USA). Briefly, 10 µL of plasma samples were diluted 1:10, incubated on a 96-well plate coated with mouse leptin antibody. After washing, a biotinylated anti-mouse leptin antibody was added. Next, the plate was washed, horseradish peroxidase-conjugated streptavidin was added. Following another washing step, a buffered 3,3,5,5′-tetramethylbenzidine solution was used to produce a color change reaction stopped after 30 min with 0.2-M sulfuric acid. Color intensity was read at 450 nm. Concentrations were calculated using a standard curve (standards run in duplicate, *R*^2^ = 0.995).

### Characterizing Herbivory on Wild Plants Using DNA Metabarcoding

To compare the wild plant species diversity and composition with which lab mice supplemented their diets, we employed a DNA metabarcoding method that involves sequencing and identifying undigested plant DNA obtained from fecal pellets (42, 43). The plant DNA in fecal pellets is likely to reflect very recent foraging activity because the half-life of ingesta in lab mice is approximately 74 min (44), meaning that <1% of contents are retained after 8 h. Thus, the resulting dietary profiles represent plants eaten over the ~2.5- to 5-h period prior to sample collection (44, 45).

Fecal samples from 26 mice (*n* = 7 HP inf, 9 LP inf, 6 HP uninf, 4 LP uninf) from the end of the study were obtained for dietary analysis. Samples were frozen on dry ice and stored at −80°C to preserve DNA prior to extraction. Total DNA from 1 to 2 pellets (~15–30 mg) per mouse was extracted using a Zymo Xpedition Soil/Fecal DNA mini kit with an extraction blank to monitor for potential cross-contamination. Using PCR, the P6 loop of the chloroplast *trnL*(UAA) marker was amplified with primers *g* and *h* (42). The PCRs were run with unique combinations of the *g* and *h* primers that had been modified with 8-nt multiplex identification (MID) tags in order to enable pooling of amplicons for sequencing using established protocols (46). These PCR products were cleaned and normalized using SequelPrep normalization plates, then pooled for sequencing in a 170-nt single-end run of the Illumina HiSeq2500 at Princeton University’s Lewis Sigler Institute following Kartzinel et al. (46).

The resulting DNA sequence data were assigned to samples of origin (i.e., demultiplexed), screened to reduce potential sequencing errors, and identified by comparison to a plant DNA reference library. The sequences were demultiplexed and primers were removed using the *ngsfilter* command in the Obitools software (47). We discarded sequences that contained ambiguous base calls (i.e., non-A, T, C, or G characters) that were <9-nt long or that had mean Illumina quality scores of <32. Unique sequences were merged and tabulated within samples to permit quantification of DNA sequence relative read abundance (RRA), a measure of the proportion of each dietary plant species in each dietary sample. Putative errors were screened by using the *obiclean* command to identify sequences that differed by 1 nt from another sequence in the same sample, but that occurred at <5% of the abundance. These sequences were removed from further analyses. Plant DNA was identified by comparison to a reference library developed using the European Molecular Biology Laboratories (EMBL) (Database release no. 130). From this archive, we extracted 35,776 unique sequences (229,430 entries) with ≤3 mismatches to the *trnL*-P6 primers *g* and *h*. Unique dietary sequences were identified by comparison to this reference database using the *ecoTag* command. Operational taxonomic units (OTUs) were identified by clustering at the >97% level using the uclust algorithm (48). To focus on abundant and well-identified plant taxa, we considered only OTUs with >80% identity to the EMBL database and those representing >5% of reads within each sample. Samples were rarefied to even sequencing depth using phyloseq (49) in R (50).

We inspected taxonomic identifications to identify imprecision that could arise from gaps or misidentified DNA sequences in the reference library. Any OTUs that exactly matched a taxon not known to occur in the region were revised to higher taxonomic levels, and the set of references matching any OTUs that were poorly identified (family or higher taxonomic levels) were scrutinized for taxonomic outliers (Table S2 in Supplementary Material). Only two plant taxa were included in the manufacturing process of the chow provisioned to lab mice in this experiment were corn (*Zea mays*) and beets (*Beta vulgaris*), and DNA from these plant products was expected to be destroyed by irradiation during the chow production process; indeed, no DNA sequences that match either of these plant species were identified in the final set of OTUs from our analysis. We calculated RRA by converting the rarefied number of reads into a proportion of reads per sample (i.e., ranging 0–1). Although RRA is not always a reliable measure of proportional dietary utilization in DNA metabarcoding studies (51), the analysis of RRA based on the *trnL*-P6 protocol employed in this study has been supported at least to the level of plant family and functional group in independent studies from different systems (46, 52, 53).

### NMR of Dietary Metabolites

Nuclear magnetic resonance spectroscopy (NMR) was used to examine dietary metabolites from a random subsample of uninfected mice (12 HP and 8 LP) with sufficient frozen fecal samples from the end of the experiment. Fecal pellets from the mice were weighed, crushed, and diluted 1:3 with phosphate buffered saline (PBS). Samples were vortexed, allowed to dissolve overnight, and re-vortexed. After centrifugation, the supernatant was decanted and brought to ca. 30 µL with PBS in a 1.7-mm OD capillary tube (New Era Enterprises, Vineland, NJ, USA). This capillary was inserted into a 5/2.5-mm OD NMR tube containing small amount of D_2_O, which served as an external lock material. Samples were analyzed on an 800-MHz Bruker Avance III HD NMR spectrometer equipped with a custom-made ^1^H/^19^F/^13^C/^15^N//^2^H cryoprobe using Topspin v.3.2. Water suppression was done using the first increment of the NOESY pulse sequence (delay–G1–90^o^–t_1_–90^o^–t_m_–G2–90^o^–acquisition) applying weak presaturation during the relaxation delay and the 100-ms mixing time period. Data processing was done offline using MNova v.11.0 (Mestrelab Research, Santiago de Compostela, Spain). After zero filling, apodization with 1-Hz Gaussian broadening, careful phase and baseline correction were applied manually. In order to compensate for variance of concentrations the spectral intensities were normalized to total integral, excluding the small region of the residual water signal. Local peak alignment was applied where necessary and possible using the inherent function in MNova. A few key metabolites were identified based on literature data (54). Out of the 20 samples, two were discarded because of technical problems (poor shimming and water suppression) to maintain statistical integrity of the residual data. The collection of 18 spectra was then exported to a spreadsheet.

### Statistical Analysis

The final sample size of this study was 80 (LP infected = 31, LP uninfected = 10, HP infected = 24, HP uninfected = 15). Several mice eluded capture for over 20 days beyond when the rest were trapped and sampled; these were excluded from statistical analyses. An additional mouse that had a severe congenital uterine defect was also excluded.

We assessed effects of diet and infection on immunity and condition. No uninfected mice had detectable IL13 concentrations, so a Kruskal–Wallis rank sum test was first used to compare infected and uninfected mice. The other cytokines (IFNγ, IL10, IL17) also had highly skewed distributions that could not be normalized. Thus, effects of diet and infection were analyzed using Kruskal–Wallis tests. Next, a general linear model (GLM) was used to test the effect of diet on log-transformed IL13 concentration among infected mice. GLMs were also used to test effects of diet and infection on log-transformed worm-specific IgG1, total IgG, and spleen weight relative to carcass weight. Using GLMs, we next tested for effects of diet and infection on mouse condition, including weight gain per day, plasma albumin concentration, plasma leptin concentration, and carcass weight. Plasma albumin and leptin concentrations were log transformed for the analysis. Diet–infection interaction terms were dropped when non-significant. To account for potential correlation structure among plasma components, we conducted a principal component analysis (PCA) of worm-specific IgG1, total IgG, albumin, and leptin. All components were log transformed and scaled prior to analysis. Next, the relationship between diet and infection status and each principal component (PC) was tested using GLMs. This PCA yielded no new insights beyond the univariate analyses described above, and results can be found in Table S1 in Supplementary Material.

To assess differences in fecal metabolites between the diet treatments, the SIMCA-P v.12.1 software package was used (Umetrics, Umea, Sweden). To determine if there was spectrum-wide variation between the diets, partial least squares-discriminant analysis (PLS-DA) was performed and orthogonal atrial least squares-discriminant analysis (OPLS-DA) were performed. Widely used in metabolic phenotyping studies, the PLS-DA analyses are better able to detect clusters than traditional PCA, and OPLS-DA provides even stronger discrimination based on known groupings (e.g., diet) (55). The DA methods were validated by calculating *R*^2^ and *Q*^2^. Prior to PLS-DA and OPLS-DA analysis, both UV and Pareto scaling were applied. UV scaling is a better choice for maintaining uniform contribution from all peaks regardless of their absolute intensity, while Pareto scaling provides access to “spectrum-like” loadings plot.

To examine morphological and behavioral changes, we first tested for effects of diet and infection on large intestine size (tissue weight/carcass weight) using a GLM. Similarly, we used a GLM to determine if time spent feeding per day in the enclosures varied with diet or infection status. Third, we examined supplemental foraging on natural plants in the enclosures using fecal DNA metabarcoding data. We tested for significant differences in the composition of plant species eaten according to diet, infection status, and leptin levels (log-transformed) using permutational MANOVA (perMANOVA) in *vegan* (56) in R. For the perMANOVA, we calculated pairwise Bray–Curtis dissimilarity metrics for each pair of samples, which ranges from 0 to 1 (from completely identical to mutually exclusive dietary compositions, respectively). For the six wild-plant families with the greatest overall RRA, we compared mean RRA between treatment groups (infected vs. uninfected and LP vs. HP diets) and across levels of leptin. Exploratory trend lines were fit to the data using generalized linear modeling. We investigated differences in RRA between genera of the legume family (Fabaceae) in more detail because this family comprised greatest overall RRA.

Lastly, we tested for differences in parasite load and weight gain, corrected for the amount of time each individual spent feeding. Among infected individuals, a non-parametric Kruskal–Wallis test was used to determine if diet affected worm count. In infected individuals, we also tested for an effect of diet on worm length (log transformed for normality) with a GLM. The amount of weight each mouse gained while in the enclosures was divided by the amount of time each spent feeding. Weight gain per minute feeding was compared between diets and infection status using a GLM. All analyses were run in R version 3.1.2.

## Results

### Dietary Quality and Infection Affected Immunity

**Figure 3:**
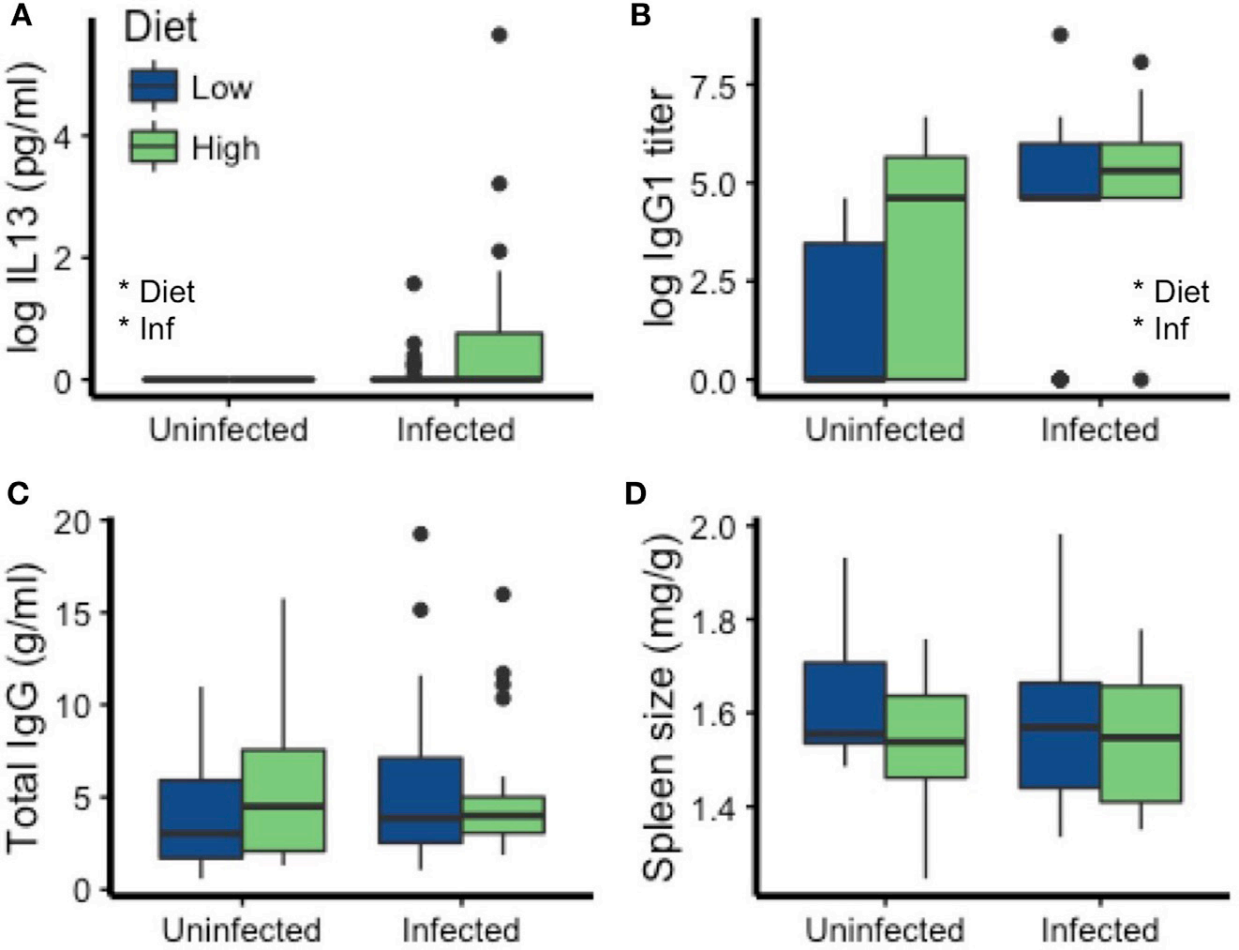
Diet and infection status affected some mediators of immunity to *T. muris* but not others. Specifically, **(A)** interleukin 13 (IL13) and **(B)** immunoglobulin G1 (IgG1) were affected by diet and infection, but not **(C)** total IgG concentration or **(D)** spleen size (weight/carcass weight). Asterisks denote significant effects of diet or infection (Inf).

Measures of immune function were affected by both diet and infection. MLN cells of the uninfected mice produced no detectable IL13 [a cytokine strongly induced by nematodes (57)] in response to stimulation with nematode antigen, making them significantly lower than the infected mice (Kruskal–Wallis χ^2^ = 7.52, df = 1, *p* = 0.0061; Figure 3A). Among infected mice, those on the high-protein diet had higher IL13 concentrations in culture supernatants than those on the low-protein diet [Est (±SE): 0.56 ± 0.25, *t* = 2.20, *p* = 0.033; Figure 3A]. The cytokines IFNγ and IL17 mediate pro-inflammatory responses, primarily to intracellular pathogens and extracellular bacteria and fungi, respectively (58, 59). IL10 mediates anti-inflammatory, regulatory responses (58, 60). Surprisingly, IFNγ, IL17, and IL10 were also higher in infected mice but did not differ between diets (Figure S1 in Supplementary Material). In mice and many other mammals, IgG1 is an important antibody class for fighting GI nematode infection (57). In addition to being higher in infected mice (*p* = 0.00006), *T. muris* specific IgG1 titers were elevated in mice eating the high-protein diet (*p* = 0.032; Table 2; Figure 3B). Total IgG concentrations did not vary with diet or infection status (all *p* > 0.33; Table 2; Figure 3C), so we cannot account for the elevated IgG1 titers observed in uninfected mice eating the high-protein diet as a correlate of high total IgG concentrations. Spleen size also did not differ significantly with infection status or diet (all *p* > 0.10; Table 2; Figure 3D).

**Table 2:** Results of general linear models testing the effects of diet (HP) and infection on immunity, condition, morphology, behavior, and costs of infection.

	Estimate	SE	*t*-Value	*p*-Value
**Log (*T. muris* IgG1 titer)**				
Diet	0.367	0.168	2.18	**0.032**
Infection	0.769	0.182	4.23	**0.00006**
**Log (total IgG g/mL)**				
Diet	0.171	0.175	0.98	0.332
Infection	0.129	0.188	0.68	0.496
**Spleen size (g/mm)**				
Diet	−0.056	0.034	−1.64	0.106
Infection	−0.022	0.037	−0.59	0.560
**Weight gain/day (g/day)**				
Diet	−0.013	0.009	1.33	0.187
Infection	−0.020	0.010	1.97	0.052
**Log (albumin mg/mL)**				
Diet	0.161	0.057	−2.83	**0.006**
Infection	0.082	0.061	−1.34	0.184
**Carcass weight (g)**				
Diet	−0.626	0.219	2.86	**0.006**
Infection	−0.341	0.236	1.44	0.154
**Log (leptin pg/mL)**				
Diet	−0.289	0.141	2.05	**0.044**
Infection	−0.018	0.151	0.12	0.904
**Large intestine size (mg/g)**				
Diet	0.56	0.41	1.37	0.176
Infection	1.03	0.44	2.34	**0.022**
**Cecum size (mg/g)**				
Diet	0.148	0.061	2.45	**0.017**
Infection	0.130	0.065	1.99	**0.050**
**Time spent feeding (min/day)**				
Diet	−13.7	3.7	−3.69	**0.0004**
Infection	−0.49	4.04	−0.12	0.904
**Weight gain/time feeding (g/day)**				
Diet	−0.088	0.265	−0.33	0.742
Infection	−0.596	0.287	−2.07	**0.042**

*Significant effects are highlighted in bold*.

### Dietary Quality Affected Nutrition and Condition

**Figure 4:**
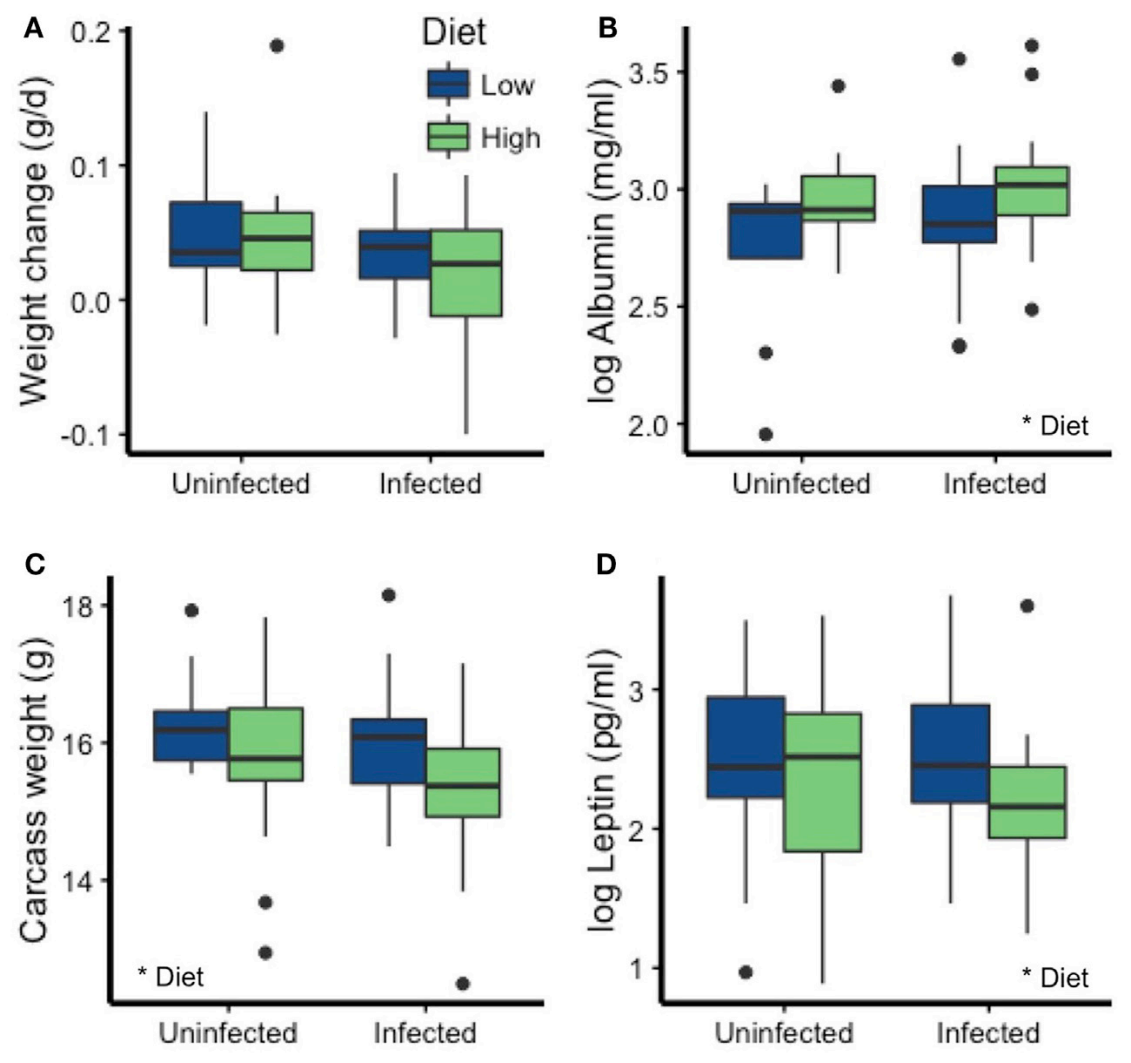
Diet, but not infection status, affected most measures of condition. **(A)** Weight change over the course of the experiment (corrected for no. of days in the enclosure) was not affected by diet or *T. muris* infection. However, the LP diet led to reduced **(B)** albumin concentration and increased **(C)** carcass weight and **(D)** leptin levels. Asterisks denote significant effects of diet.

Dietary protein affected multiple aspects of mouse nutrition and condition, whereas parasite infection did not. Although neither diet (*p* = 0.19) nor infection (*p* = 0.052) significantly affected rates of weight change (Figure 4A; Table 2), other condition measures were more sensitive. Mice on the LP diet had significantly lower albumin concentrations (*p* = 0.006; Figure 4B; Table 2) than those on the high-protein chow. The low-protein diet also led to elevated leptin concentrations, a metabolic and immune-regulatory hormone released in proportion to body fat, as well as to higher carcass weights (*p* = 0.044; Figures 4C,D; Table 2). Infection did not affect any of these other condition measures (all *p* > 0.15; Table 2).

### Dietary Quality Affected Fecal Metabolites

**Figure 5:**
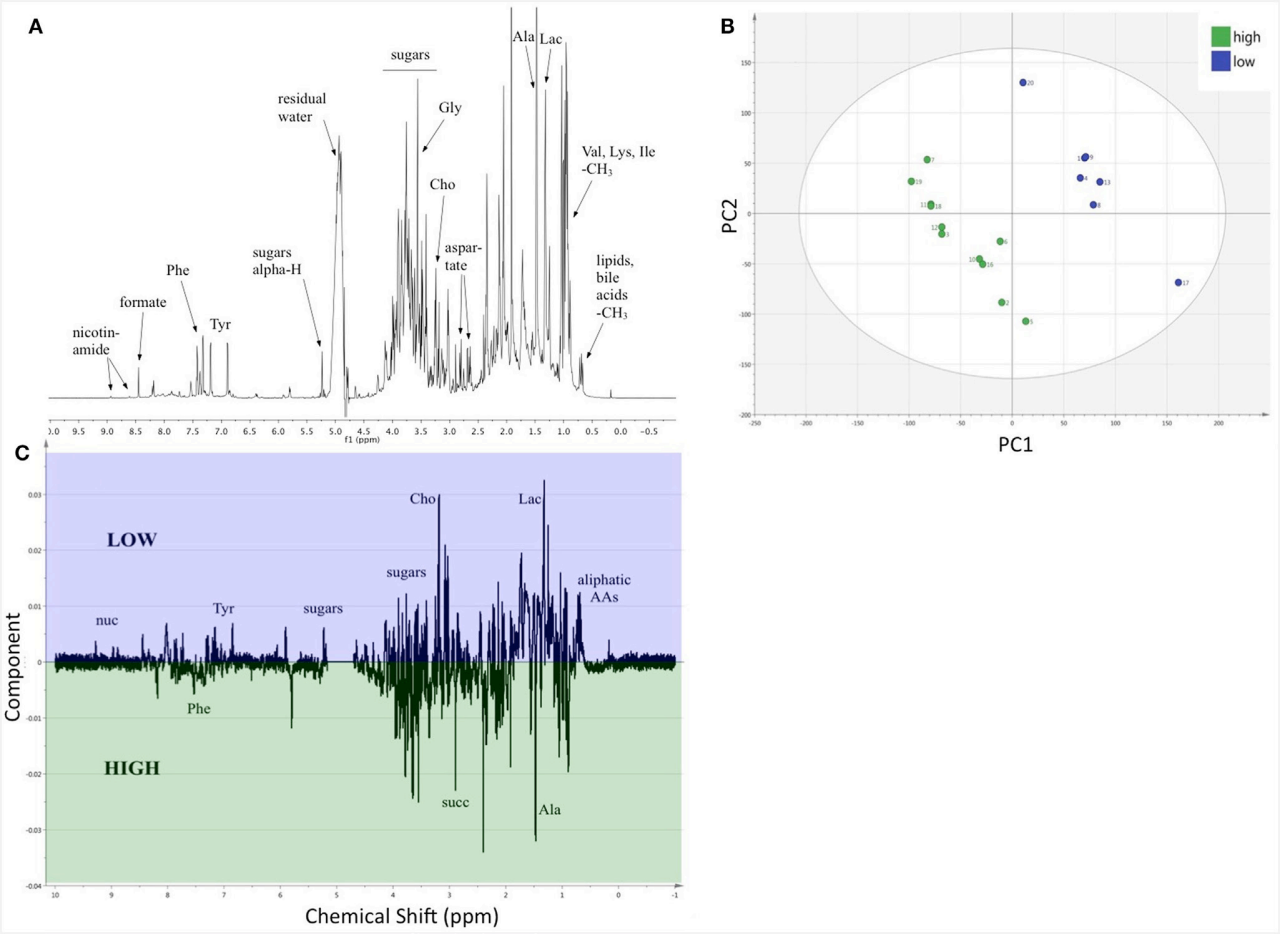
The relative composition of fecal metabolites differed between the two diets. **(A)** A representative ^1^H-NMR spectrum, the average of the 18 samples, with identification of selected metabolites. **(B)** PLS-DA scores plot (UV scaling); the subgroups of mice on HP and LP diets are clearly separated into distinct clusters. For three components *R*^2^*Y*(cum) = 0.993 and *Q*^2^(cum) = 0.533, showing decent validity of the statistics. The ellipse denotes Hotelling’s T2. **(C)** Loading data along the NMR spectrum (Pareto scaling) reveals that there are a great number of metabolites, which are present in distinct quantity in the separated clusters of samples. All the negative intensities belong to peaks of metabolites, which are present in greater quantity in the cohort on HP diet (green), while the positive intensities depict metabolites in larger concentration in the LP diet group (blue), respectively. Some tentative assignments are shown on the plot. Abbreviations: AAs, amino acids; Ala, alanine; Cho, aldehydes; Gly, glycine; Ile, isoleucine; Lac, lactones; Lys, lysine; nuc, nucleic acids; Phe, phenylalanine; Tyr, tyrosine; succ, succinate; Val, valine.

Nuclear magnetic resonance spectroscopy provides data and information on metabolites at the molecular level. The average spectrum of the 18 fecal samples, after normalization, peak alignment, and identification of some components (54), is shown in Figure 5A. PLS-DA (UV scaled) revealed distinct clustering by diet (Figure 5B), with a high proportion of variance explained by PC1 and PC2 (*R*^2^*Y* = 0.993) and decent predictability (*Q*^2^ = 0.533). The OPLS-DA (Pareto scaled) coefficient plot (Figure 5C) shows many positive and negative intensities, corresponding to metabolites in higher or lower relative concentrations in LP and HP diets, respectively. A great variety of sugars are present in the fecal extract, and aliphatic amino acids are clearly in higher abundance in the LP diet group (Figure 5C). The differences in metabolite relative abundances between the diet treatment indicates the LP diet produces clear alterations in the metabolism of the mice and/or their gut microbiota.

### Dietary Quality and Infection Altered Aspects of Morphology and Behavior

**Figure 6:**
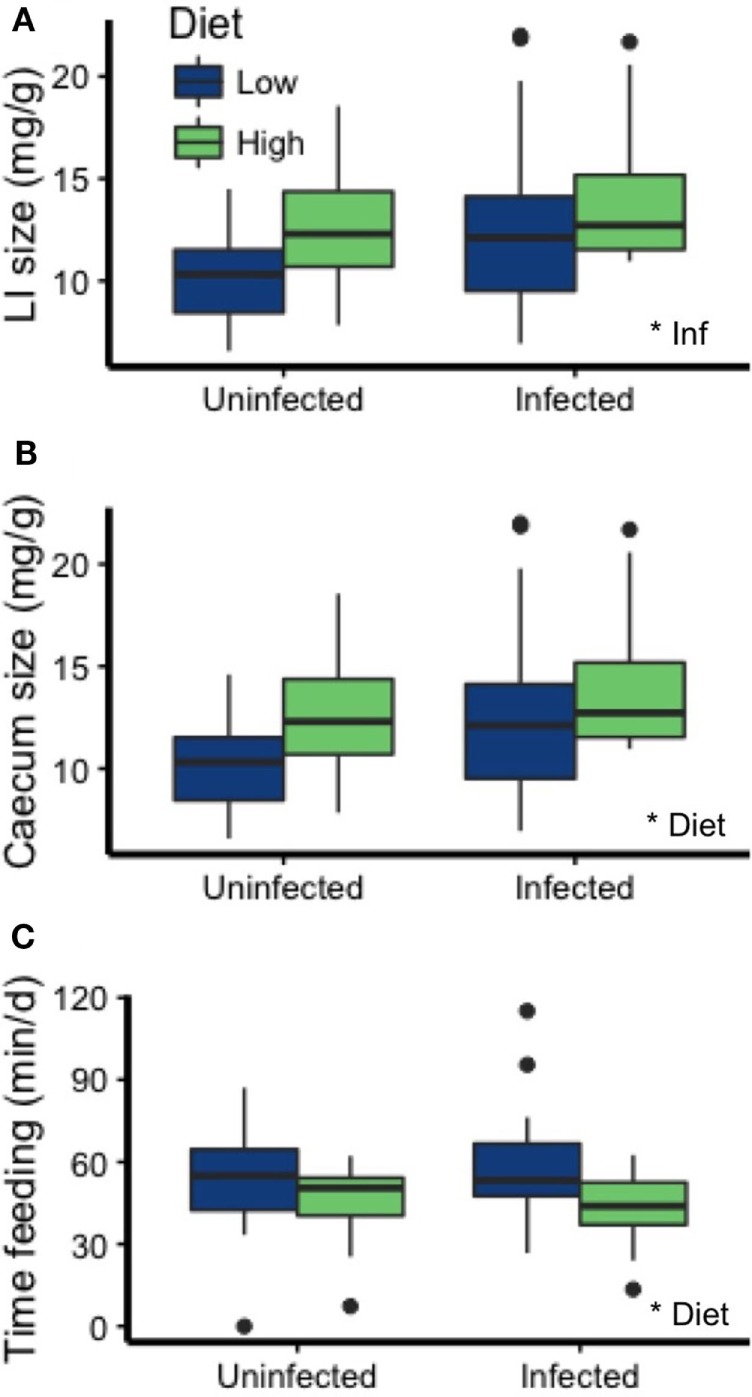
Mouse physiology and feeding behavior were affected by infection and diet. **(A)** Infection was associated with a heavier large intestine size (emptied of contents, relative to carcass weight), whereas **(B)** the HP diet was associated with a heavier cecum relative to carcass weight. **(C)** Mice on the LP diet spent more time feeding than mice on the HP diet. Asterisks denote significant effects of diet or infection (Inf).

The morphology and behavior of the mice was affected by diet and infection. First, infected mice had significantly heavier large intestines (emptied large intestinal tissue, weight relative to carcass weight) than uninfected mice (*p* = 0.022; Figure 6A; Table 2). Diet and infection status affected cecum size, with larger ceca in infected mice on the HP diet (diet: *p* = 0.017, infection: *p* = 0.050; Figure 6B; Table 2). Additionally, mice on the LP diet spent more time feeding than those on the HP diet (*p* = 0.0004; Figure 6C; Table 2).

### Herbivory on Wild Plant Species Varied among Individuals

Our DNA metabarcoding strategy yielded a total of 1,433,421 demultiplexed sequence reads of high quality, 14,521 of which were unique. After removing putative sequencing errors and picking OTUs, our cleaned-up database comprised 3,017 OTUs representing 1,296,521 sequence reads (>90% of the original). Sequence counts per sample were uneven (range = 1,648–162,180, mean = 49,702), and all were much higher than the extraction blank (*N* = 229). After removing sequences that poorly matched the reference database and that were never >5% of reads in any sample, we were left with 82% of the raw DNA sequence reads (1,167,514; of raw DNA sequence reads). These raw DNA sequence reads represented 26 plant OTUs in subsequent analyses (Table S3 in Supplementary Material). We rarefied samples to an even depth of 1,301 sequences/sample. The six most heavily utilized wild plant families (RRA > 0.05; Table S3 in Supplementary Material) included legumes (Fabaceae, cumulative RRA across all samples = 0.32), grasses (Poaceae = 0.18), wood sorrel (Oxalidaceae = 0.12), roses (Rosaceae = 0.08), violets (Violaceae = 0.07), and asters (Asteraceae = 0.07).

**Figure 7:**
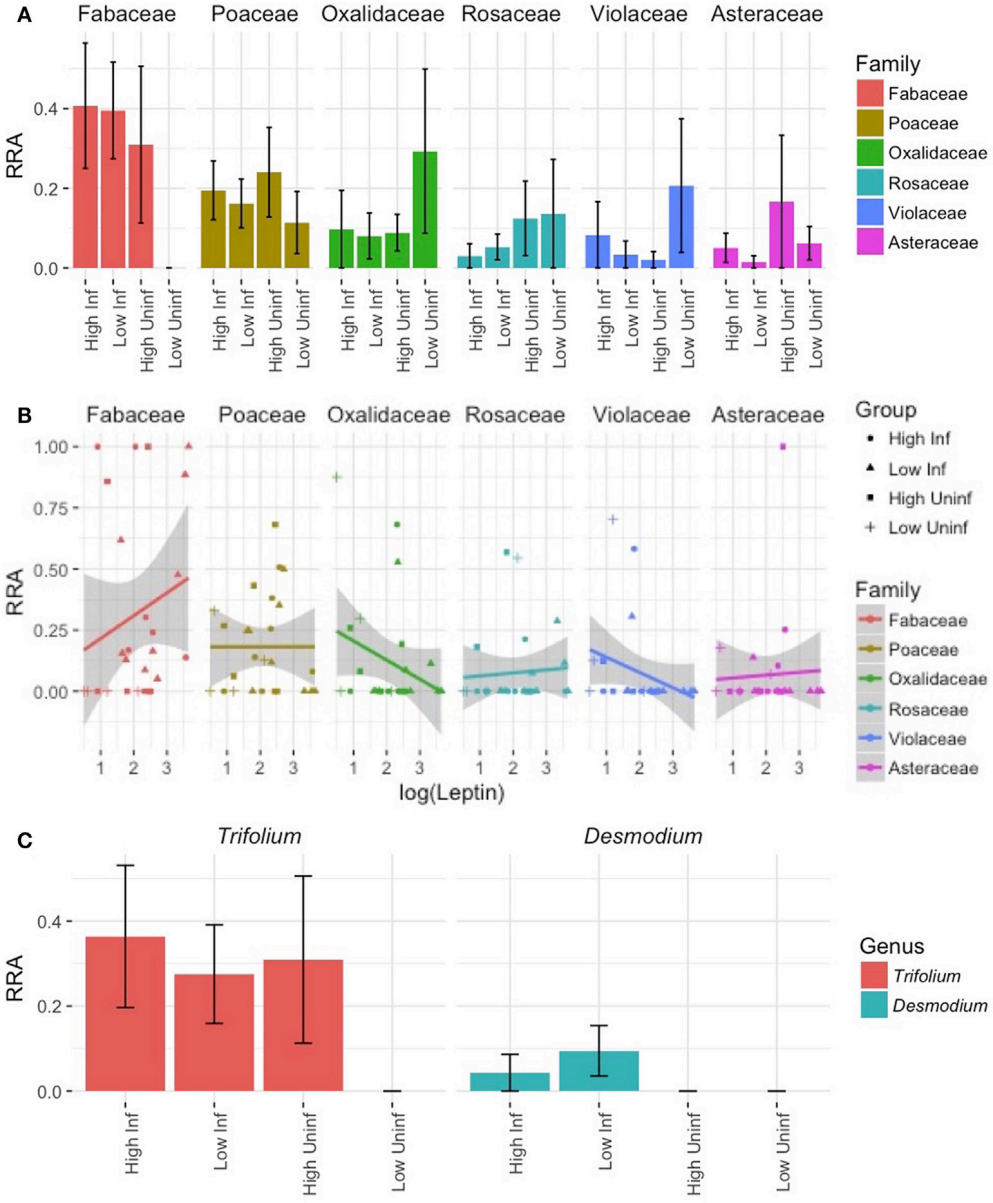
Dietary DNA metabarcoding revealing the diversity of wild plants eaten by lab mice. **(A)** The mean (±SE) relative read abundances (RRA) of plants representing the top-6 most heavily utilized families of wild plants reveal considerable dietary variation within and among treatment groups. Families are ordered according to decreasing total RRA across all samples. **(B)** The correlations between leptin, a measure of body fat, and the RRA of plant families in each sample suggest differing relationships, but none reached significance (all *p* > 0.05). **(C)** Within the family exhibiting the highest overall RRA (Fabaceae), an OTU-representing *Trifolium* (clover) was common in all but the LP-uninfected treatment and an OTU-representing *Desmodium* (beggar’s lice) was eaten only by infected mice.

Individuals varied considerably in the composition of wild plants eaten, but the overall composition of wild foods eaten did not differ between treatment groups. The composition of consumed wild plants did not differ by diet quality (perMANOVA: pseudo-*F*_1,22_ = 0.83, *R*^2^ = 0.03, *p* > 0.05) or infection status (pseudo-*F*_1,22_ = 0.96, *R*^2^ = 0.04, *p* > 0.05; Figure 7A) of the mice, at least in part reflecting the high inter-individual variation in diet composition (mean pairwise Bray–Curtis dissimilarity = 0.89). Diet compositions were more closely associated with leptin levels (pseudo-*F*_1,22_ = 1.61, *R*^2^ = 0.06, *p* = 0.06; Figure 7B), although this trend was marginally non-significant.

Nevertheless, some variation among groups in their proportional utilization of different plant types was apparent. Most strikingly, legumes (family Fabaceae) were virtually unutilized by LP-uninfected lab mice even though the mean RRA of legumes eaten by mice in other treatments ranged from ~0.3 to 0.4 (Figure 7A). Within Fabaceae, clover (*Trifolium* sp.) was proportionally more utilized by individuals assigned to all groups except the LP-uninfected group, while beggar’s lice (*Desmodium* sp.) was proportionally more utilized by infected individuals (Figure 7C). Legumes tended to have higher RRA in the diets of individuals with high leptin levels, and there was a trend of decreasing Oxalidaceae and Violaceae RRA with leptin (Figure 6B). Lab mice from the LP-uninfected treatment utilized proportionally more Oxalidaceae (mean RRA ~0.3 vs. <0.1) and Violaceae (mean RRA ~0.2 vs. <0.1; Figure 7A). These trends did not, however, reflect a significant relationship (*p* > 0.05).

### Effects of Infection Visible Despite Low-Intensity Infections

**Figure 8:**
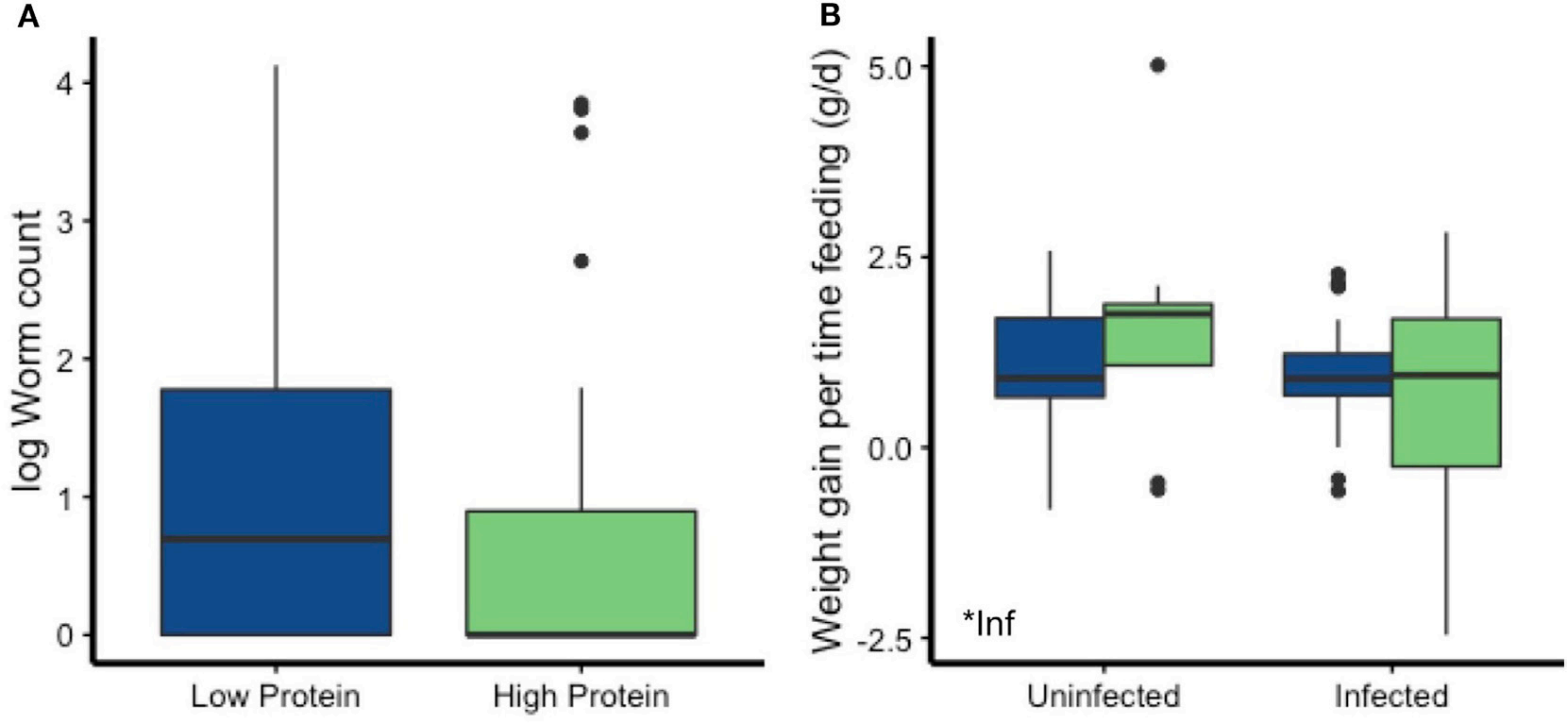
Despite there being no differences in worm counts by diet, infected mice gained less body weight than uninfected mice when corrected for time spent feeding. **(A)** Among infected mice, worm counts did not differ by diet. **(B)** Infection status affected weight gain for the amount of time individual mice spent feeding.

Perhaps due to the greater chow consumption of mice on the LP diet and increased large intestine size of infected mice, the net effects of diet and infection reveal the dynamic complexity of scaling-up this host–parasite interaction to the individual level. For example, worm counts did not differ by diet treatment (Kruskal–Wallis χ^2^ = 0.27, df = 1, *p* = 0.60; Figure 8A). Worm length also did not vary with dietary quality (*t* = 0.57, *p* = 0.57).

Costs of infection were nonetheless detectable if individual-level feeding behavior was accounted for. Although overall weight change did not vary among treatments (Figure 4A), mice on the LP diet spent more time feeding (*p* = 0.0004; Figure 6C). Examining weight gain per time feeding revealed that infected mice gained weight at a slower rate than the uninfected mice (*p* = 0.042; Figure 8B; Table 2), suggesting that they had to invest that food energy into something other than growth (e.g., the immune response) or that parasites usurped it.

## Discussion

Above all, this study shows that a broad perspective on the resource demands of parasites and immunity is needed to understand mammalian defense. In order to gain this understanding as well as insight into the ecology and evolution of host defenses in general, we must incorporate behavioral as well as physiological responses to resource limitation and infection. Using diverse data types, we discovered that within-host dynamics of infection and defense were strongly impacted by the interactions of the host with its wider environment. Most importantly, effects of infection, in terms of reduced weight gain, were only visible after accounting for variation in individual feeding behavior, highlighting the complexity of resource–immune–infection relationships at the individual scale. In the sections below, we discuss each aspect of our results in detail before returning to the broader implications in a concluding section.

### Dietary Quality and Infection Affected Immunity but Not Parasite Loads

The 6% protein (low protein) chow reduced investment in both IgG1 and IL13. The direction of the effect of dietary protein on IgG1 responses to *T. muris* E/S antigen, the predominant antibody response to primary infection (61), was difficult to predict since both higher and lower responses are reasonable given previous studies. In the lab, higher IgG1 antibody concentrations during protein restriction were documented in mice infected with *T. muris* (14). However, mice infected with the nematode *Heligmosomoides polygyrus* experience lower levels of total IgG1 (15) when fed a 3% protein diet. Interestingly, a 7% protein diet permitted total IgG1 levels indistinguishable from those in mice fed a 24% protein diet (15). Similarly, the reduction in IL13 on the LP diet was not a certainty; in a previous laboratory experiment, IL13 concentrations did not vary with dietary protein level in mice infected with *H. polygyrus* (27). Spleen size was not sensitive to dietary protein, a somewhat unexpected result given that low-protein diets reduce spleen size during *H. polygyrus* infection (15, 34). However, differences between these nematodes in their infection sites (small intestine vs. cecum) and the immune responses they typically induce in C57BL/6 mice (Treg vs. Th2) (62), could account for their different effects in protein-limited hosts.

The reductions in IgG1 and IL13 could be due to direct effects of protein limitation on T- and B-cell function, which are known to be regulated by host metabolic activity (63, 64). Indeed, trade-offs between markers of protein nutrition and worm-specific antibodies to the nematode *Teladorsagia circumcinta* were visible in a wild Soay sheep population. Moreover, these trade-offs predicted overwinter survival with nutrition increasing survival for older individuals while investment in immunity led to greater survival odds for young sheep (37). Alternatively, the elevated carcass weights and leptin concentrations of the LP mice, due to increased chow consumption, suggest they had a higher body fat content than HP mice. Food intake in uninfected mice may be regulated by dietary protein content (65). Increased chow consumption, weight, and leptin levels have also been detected in mice fed a low-protein diet in previous nematode infection-diet manipulation experiments (27, 66). Carcass weight has been posited as an indicator of tolerance during helminth infection (67), but our data suggest that compensatory feeding may disrupt that association. Obesity is associated with pro-inflammatory responses (68), which may be mediated in part by leptin (6, 69, 70). Thus, the reduced IgG1 and IL13 levels could be a consequence of the fatter LP mice shifting from Th2 responses to pro-inflammatory Th1 responses. Although it is a common finding across multiple nematodes (27, 66), it remains unknown whether protein limitation itself or increased body fat due to limitation-induced overeating drive the pro-inflammatory shift during protein limitation.

Given how integral IgG1 and IL13 are to the development of an effective Th2 response to *T. muris* infection (61, 71), it is surprising—and contrary to our initial hypotheses regarding potential relationships between diet and immunoparasitological outcome—that parasite load did not differ between the dietary treatments. IgG1 levels in uninfected HP mice were much higher than those in uninfected LP mice, which contributed to the overall effect of diet on IgG1 levels. Among infected mice, IgG1 levels were more similar across diets, offering a potential explanation for their indistinguishable worm counts. Yet, among infected mice, IL13 concentrations were over 70 times higher in mice eating the HP diet, so it is unclear why that did not translate to differences in parasite load. Across both diets, infected mice had cleared most of their parasites by the time they were sampled at the end of the study. Due to this clearance rate and the high numbers of individuals with below-detection immune responses, there was insufficient statistical power to examine individual-level variation in immunity and its relation to parasite load. The treatment-level patterns suggest that perhaps the lower concentrations of IL13 in the mice given the LP diet were also sufficient to reduce *T. muris* survival by that time point, while those on the HP protein treatment had excess expression. Alternatively, the higher IL13 concentration (generated by stimulating MLN cells with *T. muris* antigen), might indicate the strength of the memory response. Thus, mice on the HP diet might be better protected during reinfection, a pattern also seen during protein limitation and infection with *H. polygyrus* (27, 34).

The low worm counts are surprising, given the 5.5-fold higher loads observed at a similar time point in a prior experiment (mean ± SE; Leung et al.: 34.2 ± 7.7 worms, this experiment: 6.1 ± 1.9 worms) in these same enclosures with similar mouse age and weight at infection, enclosure acclimation time, experiment duration, and parasite dosing performed by the same technician (See text footnote 1). The number of dpi was a strong predictor of worm counts, which declined quickly over time. If sampled sooner, differences in burden between diet treatments may have been more detectable. However, this rate of decline was indistinguishable from the prior experiment (See text footnote 1), and thus cannot explain the overall lower worm counts.

Instead, differences in hatching ability between batches of *T. muris* eggs, differential availability or composition of supplementary forage, slightly different sampling time points and/or effects of the different brand and composition of chow could contribute to the disparity in worm counts between experiments. For example, although the chow used by Leung et al. (See text footnote 1) had a similar macronutrient composition to the HP diet in this study, the highly refined ingredients in the specialty diet might have altered the gut microbial flora, reduced *T. muris* hatching, made the intestines a less hospitable environment for *T. muris*, and/or increased the efficacy of mouse immune defenses. This is an important area of future inquiry. In any case, IgG1 and IL13 data confirm that the mice in the current study were infected for long enough to stimulate an immune defense to *T. muris*.

### Dietary Quality Affected Fecal Metabolites

In the uninfected mice, the dietary quality significantly altered the nutrient environment within the mouse; in infected hosts, *T. muris* would likely experience similar nutrient alterations. Estimating cecum nutrient content from those in feces using NMR revealed that sugar metabolites varied greatly between diets, and amino acids like phenylalanine and alanine were higher in the HP group. This is not surprising given that the difference in protein between the treatments was compensated with a higher percentage of carbohydrates to achieve equal calorie density. Relative abundances of tyrosine, which is involved in the regulation of immune signaling pathways (72), and the aliphatic amino acids tended to be higher among mice on the LP diet. This preliminary exploration demonstrates that NMR metabolite analysis is a powerful, non-species-specific tool for examining host nutrition. Further analysis can include 2D NMR and spiking to identify additional metabolites, quantification of absolute concentrations using reference samples [PULCON method (73)], and metabolic pathway analysis [STOCSY (74)]. Despite differences in the nutrient environment revealed by NMR, *T. muris* counts and length did not differ between the dietary treatments. *T. muris* may feed on intestinal tissues and secretions (75, 76), rather than host blood or ingesta. *T. muris* also strongly affects, and is affected by, the host intestinal microbiome (76–78), so any changes in metabolic profile that alter microbiome composition could potentially have stronger effects on *T. muris* hatching, development, and survival. Future work could explore how host gut microbiome and metabolites effect *T. muris* infection success, and in turn, how they are affected by helminth infection.

### Dietary Quality and Infection Altered Aspects of Morphology and Behavior

Our data support the hypothesis that mice attempted to compensate for differences in the protein composition of the chow by altering their physiology and behavior. Mice spent 30% more time eating the LP diet per day, a significant time investment that could also come with increased predation risk in fully natural settings (79). While this investment could partially close the gap in protein acquisition between treatments, consumption would need to be 400% higher in the LP treatment to achieve similar protein levels to individuals on the HP diet. However, dietary protein does not affect host metabolic rate (16), so maintenance costs (Figure 1) are likely similar between treatments. Insects were also present in the enclosures and diet metabarcoding tools could be used in future studies to examine if, and to what degree, lab mice are able to utilize them as a food source. Albumin was reduced on the LP diet, revealing protein limitation within those hosts. Albumin’s primary role is maintaining osmotic pressure in the blood (80), and, in wildlife, lower levels can reflect costs of reproduction (81) and indicate reduced survival probability in wildlife (37).

Interestingly, the choice of supplemental wild forage was marginally associated with differences in leptin concentrations. Leptin concentrations tended to be higher in animals that ate proportionally more plants in the legume family, Fabaceae, which includes clover (*Trifolium* sp.) and beggar’s lice (*Desmodium* sp.). These plants have a higher protein content and are widely known to be good forage for livestock and wildlife (82), even increasing sheep weight gain by an average of 40% compared with feeding on ryegrass alone (83). Thus, behavioral compensation for immunological or infection costs may explain the trend toward higher consumption of these plants in infected mice. Conversely, lab mice that ate proportionally more plants in the families Oxalidaceae (*Oxalis* sp.; wood sorrel) and Violaceae (Viola sp.) tended to have low leptin levels. Mice consumed similar amounts of chow in the enclosures (g chow/g mouse) as they did in the laboratory setting, so wild plants probably do not represent a replacement food source for most individuals. We cannot quantify the *amount* of wild plant matter eaten by mice in the different treatments using DNA metabarcoding, but these emerging trends are suggestive of compensatory foraging behaviors worth further investigation.

Infected mice found physiological ways to compensate for the costs of *T. muris* infection, rather than following our alternate hypotheses of increased foraging or infection-induced inappetence. The increased cecum size of infected individuals could be a consequence of parasite manipulation to create more habitat space, but the increase in large intestine size with infection is more difficult to explain. Large intestines, emptied of contents and relative to body size, were over 10% heavier in infected mice. This additional weight was not due to the worms themselves, which were located in the cecum. In the average size mouse, this difference translates to a 15-mg increase in colon weight. Hosts could increase colonic tissues to enhance water balance, nutrient reabsorption, or house more commensal microbes to aid in digestion. Similarly, *H. polygyrus*, which resides in the small intestine, is associated with increased investment in small intestine tissues (67). An influx of immune cells or an increase in gut microbe communities could also contribute to these differences; this hypothesis could be examined histologically in future studies. However, given estimates of approximately 1 pg per *E. coli* cell (84) and 2.2 pg per lymphocyte (85), they cannot fully account for the increase in colon size in *T. muris*-infected mice.

To some degree, infected mice may also supplement their food intake with clovers. This trend was driven by a lack of beggar’s lice in feces of uninfected mice and an absence of clover in LP-uninfected mice. Plants in the Fabaceae family, including clovers and beggar’s lice, tend to be high in protein (82), and that could help hosts either resist infection by providing more resources for immune defense or tolerate infection by compensating for costs of infection and immune defense. A larger sample size, particularly of LP-uninfected mice, would help elucidate the efficacy and generality of this potential behavioral compensation mechanism. At this stage, however, we can conclude that infected mice gained less weight per minute of chow feeding than uninfected mice, despite increased large intestine size and use of potentially high-protein wild forage. This in turn suggests that their compensation for the costs of infection (e.g., parasite resource theft, elevation of immune defenses, tissue repair, etc.) was incomplete.

## Conclusion

Ultimately, biomedical and evolutionary immunology both aim to explain how resource costs of immunity and infection at the cellular level scale up to the whole organism embedded in its natural environment. Seeking such an explanation—and indeed bridging from biomedical to evolutionary immunology—requires altered experimental designs that allow organisms to modify both their behavior and physiology in response to infection. An experimental approach is critical given the plausibility of alternate hypotheses (e.g., increased or reduced investment in immunity with a high-protein diet; increased or reduced foraging in response to parasite infection). Laboratory mice in semi-natural enclosures such as those studied here provide such an opportunity. The enclosures provided an environment with natural thermal regimes and space for activity (e.g., foraging, digging burrows) that could generate stronger energetic demands, and therefore stronger trade-offs than under typical laboratory conditions. Given the importance of gut microbiomes in *T. muris* infection (76–78) and in nutrition–immune interactions in general (19, 86, 87), the more diverse gut microbiomes generated by the enclosures (See text footnote 1) likely provide more realistic results than laboratory settings would. Additionally, this system shows potential for future studies of microbiome-helminth-diet interactions pertinent to the increasing rates of diseases linked to nutrition and immune dysregulation in developed nations (e.g., diabetes, obesity, autoimmune disorders). Our custom-built feeding monitoring system and dietary DNA metabarcoding allowed detection of compensatory feeding on chow and wild plants, respectively, that otherwise masked effects of infection.

By investigating lab mice, we were able to utilize a large suite of physiological and immunological tools, which proved useful given their variable responses to protein and infection. Much work remains to deploy such tools and new experimental designs to definitively dissect the mechanisms of the host–parasite interaction. In semi-natural enclosures, we observed high inter-individual variation that reduces statistical power and, therefore, requires much larger sample sizes than traditional laboratory studies. For example, the relationship between infection and weight gain was just above the significance threshold (*p* = 0.052), but our power to detect an effect with total sample size of 80 individuals was only 0.45, far below the ideal power of 0.8. Thus, although we failed to detect an effect of infection on weight gain, we cannot conclude that *T. muris* does not affect mouse weight gain. Indeed, once we corrected for variation in weight gain due to time spent feeding, an effect of infection on weight gain was detectable (*p* = 0.042). Additionally, to prevent uncontrolled disease transmission and “contamination” of the enclosures with parasite eggs, we ended the experiment before *T. muris* could develop into adults, which may be a more energetically costly parasite life-stage. Longer term studies and those with trickle infections (i.e., small doses over time) will provide additional insight into this host–parasite interaction. Finally, variation in individual movement and thermoregulatory behavior is difficult to monitor in the enclosures, but may contribute to overall energy budgets and weight gain. With some weatherproofing alterations, remote activity monitoring systems such as those developed to study the activity of barn mice (88), plus temperature-sensing chips, could be a useful addition to such field enclosure studies.

Nonetheless, examining the interactions among diet, nutrients, immunity, and parasites in a realistic context revealed the central role of feeding behavior in infection outcomes and the complexity of interactions among environmental resources and within-host dynamics. Future experiments must therefore account for behavioral heterogeneity among individuals if we are to elucidate costs of parasitism and defense. Moreover, in the wild, altering feeding behavior in response to infection is a strategy available to individuals, but it may come with costs in terms of energy spent foraging, predation risk, and less time available for other behaviors (e.g., reproduction). Housing laboratory mice in outdoor enclosures provided new insights into the resource costs of immune defense to helminth infection and how hosts modify their feeding behavior to compensate for those costs.

## Ethics Statement

This research was conducted in accordance with animal care protocols approved by the Princeton University Animal Care and Use Committee (Protocol no. 1982-14).

## Author Contributions

SB and AG designed the study, with intellectual contributions from CC and AL. SB, CH, and AG carried out the experiment and performed immune and condition measurements. QC and RG designed and built the feeding monitoring system. Diet metabarcoding was performed by CH and TK, and analyzed by SB and TK. NMR was performed and analyzed by IP. SB analyzed the data (unless otherwise noted) and wrote the manuscript. All coauthors provided editorial feedback.

## Conflict of Interest Statement

The authors declare that the research was conducted in the absence of any commercial or financial relationships that could be construed as a potential conflict of interest.

## Footnotes

^1^Leung JM, Budischak SA, Chung HT, Hansen C, Bowcutt R, Neill R, et al. The shock of the new: rapid environmental effects on gut nematode susceptibility in re-wilded mice. Under review.

## Supplementary Material

Figure S1 Interferon gamma (IFNg), interleukin 10 (IL10), and interleukin 17 (IL17) concentrations were higher in infected mice than uninfected mice (Wilcoxon tests; IFNg: ***W*** = 506, *p* = 0.046, IL10: ***W*** = 483, *p* = 0.010, IL17: ***W*** = 441, *p* = 0.0036), but did not vary with diet (IFNg: ***W*** = 843, *p* = 0.67, IL10: ***W*** = 929, *p* = 0.13, IL17: ***W*** = 899, *p* = 0.28).
