# Supplementary material for: Feeding Immunity: Physiological and Behavioral Responses to Infection and Resource Limitation
Source: Front Immunol. 2018 Jan 8;8:1914. doi: 10.3389/fimmu.2017.01914 (PMC5766659; doi:10.3389/fimmu.2017.01914)
Supplement: Supplementary file 1 [file Table_1.DOCX]

Supplementary Table 1. Principal component analyses of serum proteins.

|  | **PC1** | **PC2** | **PC3** | **PC4** |
| --- | --- | --- | --- | --- |
| Std. Dev. | 1.077 | 1.019 | 1.003 | 0.892 |
| Prop. Variance | 0.290 | 0.260 | 0.252 | 0.199 |
| Cum. Prop. | 0.290 | 0.550 | 0.801 | 1.000 |
| **Rotation** |  |  |  |  |
| *T. muris* IgG1 titer | 0.415 | -0.087 | -0.815 | 0.395 |
| Albumin (mg/ml) | 0.641 | 0.476 | -0.016 | -0.602 |
| Leptin (ng/ml) | 0.600 | -0.104 | 0.580 | 0.541 |
| Total IgG (g/ml) | -0.238 | 0.869 | -0.003 | 0.434 |
|  | **Estimate** | **Std. Error** | **t value** | **p value** |
| **PC1** |  |  |  |  |
| Diet | 0.26 | 0.25 | 1.07 | 0.29 |
| Infection | 0.53 | 0.26 | 2.03 | 0.046 |
| **PC2** |  |  |  |  |
| Diet | 0.47 | 0.23 | 2.02 | 0.048 |
| Infection | 0.20 | 0.25 | 0.79 | 0.43 |
| **PC3** |  |  |  |  |
| Diet | -0.69 | 0.21 | -3.28 | 0.002 |
| Infection | -0.81 | 0.22 | -3.66 | 0.0005 |
| **PC4** |  |  |  |  |
| Diet | -0.35 | 0.20 | -1.70 | 0.093 |
| Infection | 0.23 | 0.22 | 1.05 | 0.30 |
