# Supplementary material for: Feeding Immunity: Physiological and Behavioral Responses to Infection and Resource Limitation
Source: Front Immunol. 2018 Jan 8;8:1914. doi: 10.3389/fimmu.2017.01914 (PMC5766659; doi:10.3389/fimmu.2017.01914)
Supplement: Supplementary file 4 [file Data_Sheet_1.DOCX]

Supplement – Appendix 1

*Feeding behavior monitoring system*

Individual-level feeding behavior was assessed using a custom-built monitoring system based Radio Frequency IDentification tags (RFID; 8 mm x 1.4 mm FDX-B "Skinny" PIT Tag, Oregon RFID, Portland, OR, USA) that were injected subcutaneously. When in the vicinity of an antenna, the RFID tag emits a unique string of numbers that serves as an individual’s identifier (i.e. RFID number). The monitoring system is separated into two main parts: a weatherproof hardware box, the Feeding Event Tracking Apparatus (FETA) with RFID antenna, that is deployed in the field; and a computer-based system housed in the laboratory, the Event Acquisition and Reporting System (EARS), that automatically compiles the data produced by the FETA (RFID number and timestamp). By placing two FETA boxes in sequence (Fig 2a), the order in which the two antennae detect a mouse can be used to determine directionality of movement, and thus where the mouse spent the previous time interval (i.e. inside or outside feeder).

Inside the FETA box, a RFID antenna coil, wrapped in foam for protection, is fitted around PVC tubing to maximize the detection of mice walking through the FETA (Fig 2b). The antenna is connected to a RFID reader (FDX-B/HDX RFID Reader, Priority 1 design, Australia). The RFID reader transfers the data to a cloud-connected WiFi micro-controller so the data can be accessed remotely. All components are mounted on miniature circuit boards, and power is provided by a 12V power adapter, connected to a 5V voltage regulator to ensure constant electricity supply to the RFID reader and the Particle microcontroller. Heat sinks are placed on the RFID reader and the voltage regulator (Fig 2b).

Two generations of controllers were used, Particle Core and Particle Photon (Particle, San Francisco, USA). The microcontroller runs FETA code, which simply broadcasts an event, which consists of a time stamp, the name of the FETA observing the mouse, and the RFID code it has read, every time an RFID tag is encountered. In the absence of any reading, the FETA code includes a redundancy that triggers an automated check-in message of the microcontroller every 15 minutes allowing to check that the system remains live at all times. We use Particle's cloud to broadcast and listen for events.

The FETA is deployed in the field, and as such needs to be fully weatherproof. A 2” C-type outdoor conduit box was therefore used to house the electronics, and to surround a 1.25” PVC tube that allows the mice access to the feeder. To ensure weatherproofing, the junction boxes are equipped with an O-ring seal and the PVC tubing is attached to the box using nontoxic aquarium sealant. The power supply cables are run through the sealant. Two silica packets are added in each box to absorb any remaining moisture as an additional precaution. The power cord was be encased in ½” flexible electrical tubing to prevent damage.

EARS is housed in the laboratory and is designed to constantly scan the Particle cloud for events broadcasted by FETA. It consists of a Raspberry Pi computer connected to the Particle cloud. When an event is broadcasted by FETA, EARS recovers this message, parses its content, and publishes the result to an online Google Sheet spreadsheet. The system is designed so that the spreadsheet is updated with the FETA box number, the RFID number (or an automated check-in message), and the timestamp of the event.

In addition, the Raspberry Pi runs a Routine Analysis Tool (RAT) automatically every hour. It can also perform basic analytics, including determining the most recent time each mouse fed and how many times it was observed during the previous 24 hrs. RAT also downloads all the current data and makes a backup.

The code run by the FETA is written in C++. EARS and RAT are coded in Python 2.7. All code will be made available on Github.


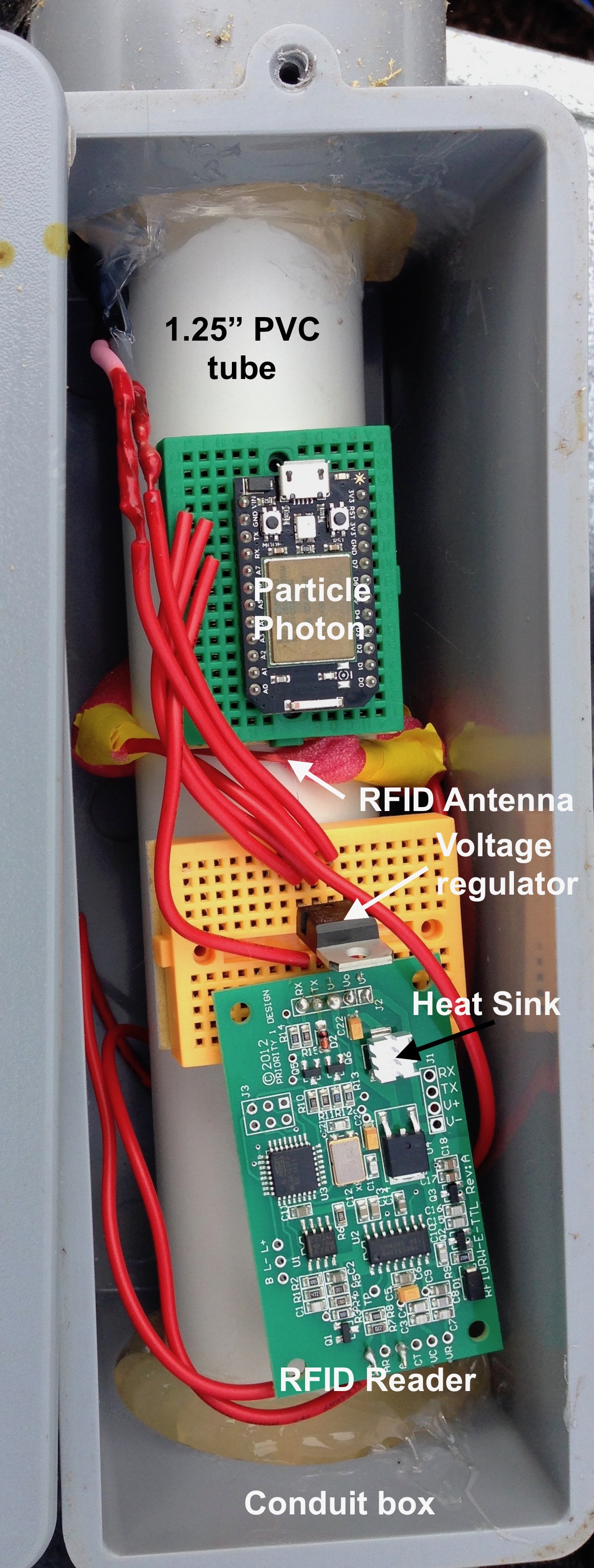


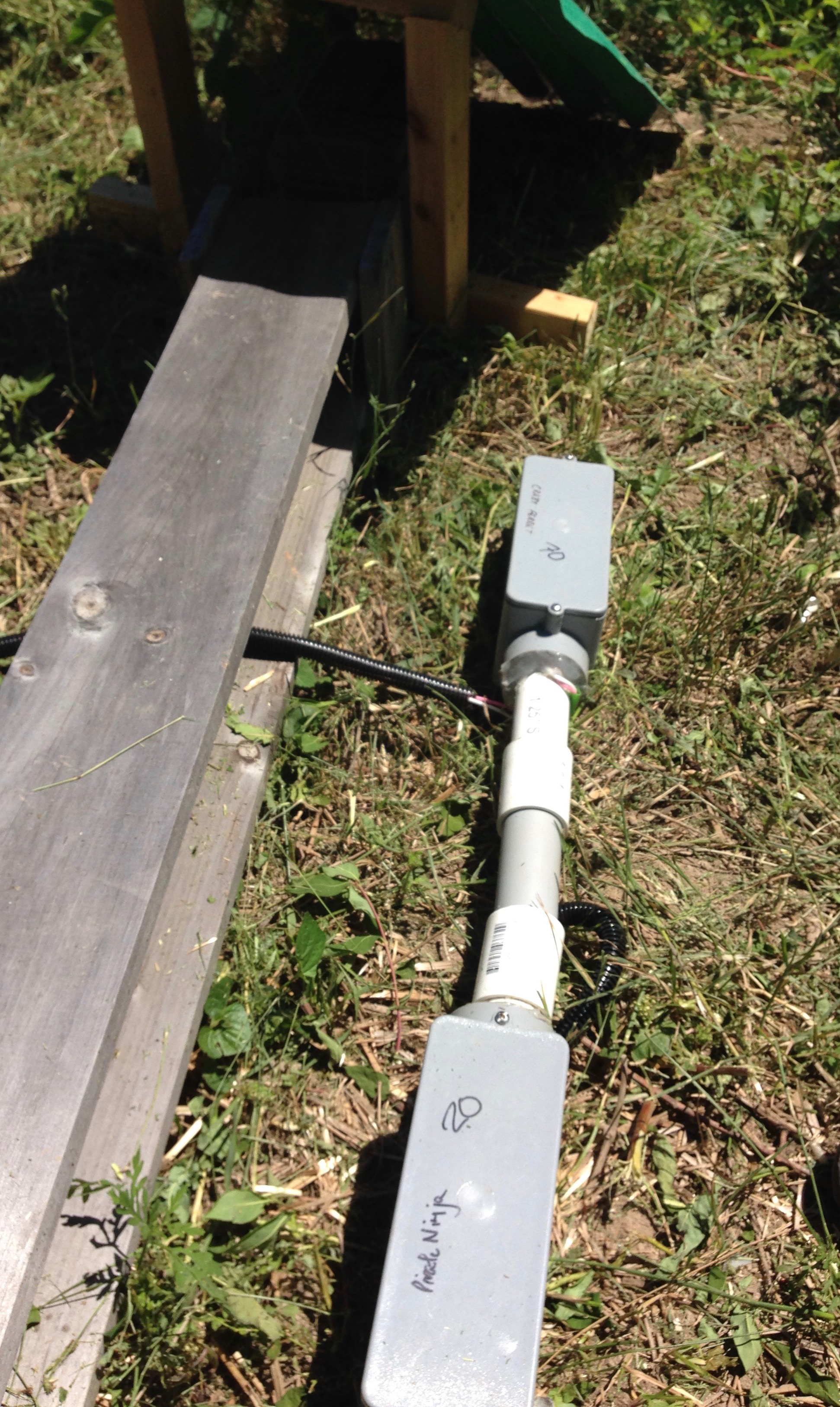


a)

b)

a) Individual feeding behavior was monitored by placing two custom-built Feeding Event Tracking Apparatuses (FETA) in series, leading into the chow hopper. b) Inside the FETA, a radio frequency identifier (RFID) antenna and microchip were connected to a Particle Photon that broadcasts mouse movement to a recording and reporting system (EARS).
