# Supplementary figures and images for: Feeding Immunity: Physiological and Behavioral Responses to Infection and Resource Limitation
Source: Front Immunol. 2018 Jan 8;8:1914. doi: 10.3389/fimmu.2017.01914 (PMC5766659; doi:10.3389/fimmu.2017.01914)

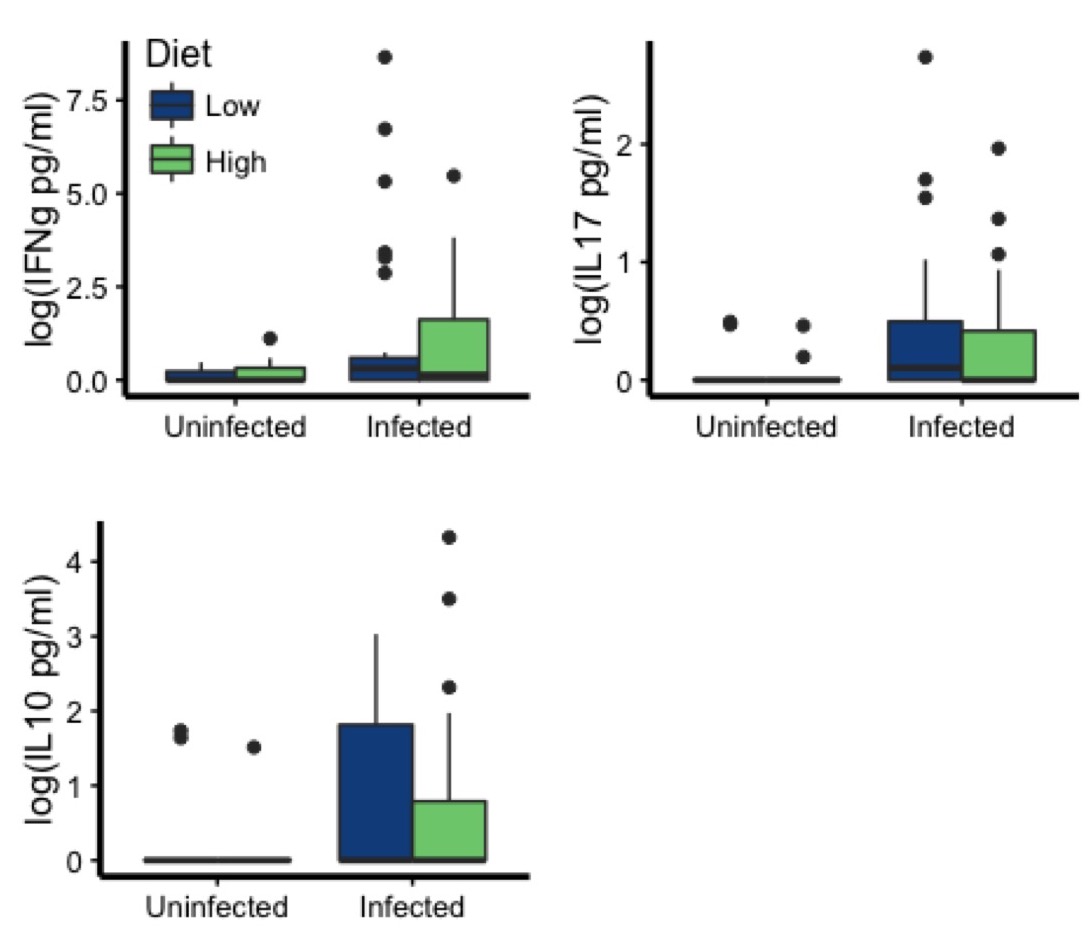

Supplement: Figure S1 — Interferon gamma (IFNg), interleukin 10 (IL10), and interleukin 17 (IL17) concentrations were higher in infected mice than uninfected mice (Wilcoxon tests; IFNg: W = 506, p = 0.046, IL10: W = 483, p = 0.010, IL17: W = 441, p = 0.0036), but did not vary with diet (IFNg: W = 843, p = 0.67, IL10: W = 929, p = 0.13, IL17: W = 899, p = 0.28). [file Image_1.JPEG]
